# Cerebral FDG-PET scanning abnormalities in optimally treated HIV patients

**DOI:** 10.1186/1742-2094-7-13

**Published:** 2010-02-14

**Authors:** Åse B Andersen, Ian Law, Karen S Krabbe, Helle Bruunsgaard, Sisse R Ostrowski, Henrik Ullum, Liselotte Højgaard, Annemette Lebech, Jan Gerstoft, Andreas Kjær

**Affiliations:** 1 Department of Infectious Diseases, Rigshospitalet, University of Copenhagen, Copenhagen, Denmark; 2 Department of Clinical Physiology, Nuclear Medicine and PET, Rigshospitalet, University of Copenhagen, Copenhagen, Denmark; 3 Centre of Inflammation and Metabolism, Rigshospitalet, University of Copenhagen, Copenhagen, Denmark

## Abstract

**Background:**

The long-term neurological consequences of HIV infection and treatment are not yet completely understood. In this study we examined the prevalence of cerebral metabolic abnormalities among a cohort of neurologically intact HIV patients with fully suppressed HIV viral loads. Concomitant analyses of circulating brain derived neurotrophic factor (BDNF) were performed to correlate these abnormalities with potential signs of neuro-regenerating/protective activity, and concomitant analyses of circulating tumour necrosis factor (TNF) α, interleukin (IL) 6, and soluble urokinase plasminogen activator receptor (suPAR) were performed to correlate these abnormalities with potential signs of neurodegenerative processes.

**Methods:**

The study population consisted of HIV-positive patients known to be infected for more than 5 years and on antiretroviral (ARV) treatment for a minimum of three years with no history of virological failure, a CD4 count above 200 × 10^6 ^cells/l and no other co-morbidities. The distribution of the regional cerebral metabolic rate of glucose metabolism was measured using fluorine-18-flourodeoxyglucose positron emission tomography (FDG-PET) scanning. The PET scans were evaluated for individual pathology using Neurostat software and for group pathology using statistical parametric mapping (SPM). Circulating levels of BDNF, TNF α, IL-6 and suPAR were measured by ELISA techniques.

**Results:**

More than half (55%) of the patients exhibited varying severities of mesial frontal reduction in the relative metabolic rate of glucose. Compared to healthy subjects, the patients with abnormal FDG-PET scanning results had a shorter history of known HIV infection, fewer years on antiretroviral therapy and higher levels of circulating TNF α and IL-6 (*p *= 0.08).

**Conclusion:**

A large proportion of optimally treated HIV patients exhibit cerebral FDG-PET scanning abnormalities and elevated TNF α and IL-6 levels, which may indicate imminent neuronal damage. The neuroprotective effect of early ARV treatment should be considered in future prospective follow-up studies.

## Background

Human immunodeficiency virus (HIV) 1 invades the central nervous system during primary infection. In some cases, patients with acute HIV infection may present with symptoms of aseptic meningitis, and pleocytosis of the cerebrospinal fluid CSF is a frequent finding among HIV patients [1]. Before the era of combined active antiretroviral (ARV) therapy, a substantial number of those patients who survived despite low levels of CD4-positive lymphocytes developed AIDS dementia complex (ADC) [2]. ARV treatment has dramatically changed the life expectancy of HIV-infected patients, and ADC is these days a rare diagnosis if patients are adherent to treatment [3]. However, a number of reports have described an apparent increase in prevalence of HIV-associated neurocognitive impairment [2,4]. The clinical definitions of the various HIV-associated conditions affecting the central nervous system are not clear and potential pathogenic mechanisms are not well understood [5]. Overlapping or common pathogenic mechanisms, as those associated with other neurodegenerative conditions, have been suggested. In a small pilot study, we found that a surprisingly large fraction of optimally treated HIV patients show minor abnormalities in the relative metabolic rates of glucose metabolism in brain as visualized by fluorine-18-flourodeoxyglucose positron emission tomography (FDG-PET) scanning [6]. These abnormalities resemble signs of imminent neurodegeneration [7,8]. The aim of this study was to perform cerebral FDG-PET scanning on a larger number of patients, and to correlate potential abnormalities with levels of circulating pro-inflammatory cytokines that have been associated with neurodegenerative conditions [9-11], and with a potential indicator of neuro-regenerative/protective activities: brain derived neurotrophic factor (BDNF) [12,13].

## Methods

Study participants were recruited from an outpatient clinic at a university hospital in Copenhagen, Denmark. Around 1300 HIV patients are registered in the clinic and around 80% receive ARV treatment. The treatment guidelines are comparable to internationally accepted recommendations and the treatment is free of charge for the patients.

*Inclusion criteria *included age between 25 and 70 years and verified HIV infection for at least 5 years. Patients should have received ARV treatment for a minimum of 3 years with no virological failure. Virological failure was defined as HIV RNA >400 copies/ml measured at two consecutive samplings with a time spacing of at least two weeks after having been fully suppressed earlier. The CD4 count should be above 200 × 10^6 ^cells/l. The study was a sub-study of a cohort of 100 patients enrolled earlier [6]. The patients were enrolled sequentially for PET scanning as they appeared in the outpatient clinic.

*Exclusion criteria *were anaemia (defined as a haemoglobin concentration more than 1 mmol below the lower limit of the normal range), hepatitis C infection, elevated levels (>4 times the upper limit of normal) of liver-derived transaminases, chronic renal failure (carbamide levels greater than 2× the upper limit of normal), hypo- or hyper-thyroidism, focal neurological disease, I.V. drug abuse, alcohol addiction, and other obvious or serious co-morbidities. Also excluded were persons unable to understand, read, and write the Danish language, and pregnant or lactating women.

The following data were recorded from patient files: age, sex, employment status, number of years of known HIV infection, mode of HIV transmission, nadir CD4 count, maximum HIV RNA load, number of years on antiretroviral therapy, number of years the patient received "*d-drugs*" i.e. stavudine and/or didanosin, present ongoing treatment regimen, C-reactive protein level, and thyroid-stimulating hormone level.

**Serological analyses **were performed on plasma samples, which had been stored at -80°C. Venous blood samples were drawn into ethylenediaminetetraacetic acid- (EDTA-) stabilized vacutainer^® ^tubes (BD-Denmark, Brøndby, DK) and kept on wet ice. Plasma was recovered by centrifugation within 30 minutes. BDNF levels were measured by ELISA (R&D Systems, Minneapolis, MN, USA). After thawing, samples were centrifuged at 10000 × *g *for 10 minutes at 4°C for removal of platelets. Samples were analysed in duplicate and mean concentrations were calculated.

The levels of TNF-α and IL-6 were measured in commercially available enzyme-linked-immunosorbent-assay (ELISA) based kits (R&D systems Europe Ltd., Abingdon, UK). suPAR levels were determined as described earlier by a double-sandwich ELISA based on a murine monoclonal anti-suPAR antibody as the catching antibody and a polyclonal rabbit antibody served as the detecting antibody [14,15]. Colour reaction was obtained after incubation with alkaline phosphatase-conjugated anti-rabbit IgG (Sigma-Aldrich, Schnelldorf, FRG) using p-nitrophenyl phosphate as the chromogen. All measurements were performed in duplicate using either un-diluted (TNF-α and IL-6) or diluted 1:10 (suPAR and BDNF) sample. Serial dilutions of control antigen and blanks were included on each plate.

### Cerebral FDG-PET scanning

Patients fulfilling the inclusion criteria were invited to have a cerebral FDG-PET scan. Recruitment was stopped when 40 patients were enrolled. Two patients were excluded after the scanning because they exhibited focal lesions and it was verified that they had in fact been treated for cerebral toxoplasmosis at another hospital. The distribution of relative regional cerebral glucose metabolic rate (rCMRglc) was measured using 18-fluoro-deoxyglucose (FDG) PET scanning. PET scans were performed with an eighteen-ring GE-Advance scanner (General Electric Medical Systems, Milwaukee, WI, USA) operating in 3D-acquisition mode, producing 35 image slices with an interslice distance of 4.25 mm. The total axial field of view was 15.2 cm with an approximate in-plane resolution of 5 mm [16]. Each patient received an intravenous bolus injection of approximately 200 MBq 18-FDG while resting in the supine position with eyes covered and noise level kept at a minimum. After 30 min the patient was placed in the scanner, and the head fixed to restrict movements. A 10 min transmission scan was performed for attenuation correction followed by a 10 min 3-D emission scan. PET images of the FDG distribution were reconstructed using a 4.0 mm Hanning filter transaxially and an 8.5 mm Ramp filter axially.

### Image analysis

Two strategies were used to evaluate the images designed to identify pathological traits on an individual level as well as for the group. The group analysis enables exploration of the images for common features, but is a less powerful approach in a setting dominated by heterogeneous findings. Initially each individual PET scan was analysed using Neurostat, that allows direct visualization of the extent and topography of FDG uptake abnormalities [17]. This procedure involves the reconstruction of the images with an 8-mm Hann filter, warping them to a standard stereotactic space and projecting globally normalized activity unto the cerebral surfaces. The surface projects are subsequently compared voxel-by-voxel to 3 cohorts of age-matched groups of healthy subjects depending on the patients' age (19-34 years, 30-60 years, and 55-90 years). This control material was not recruited at our institution, but supplied with the Neurostat software consisting of neurologically intact healthy volunteers [17]. The HIV status of this control group is not known. Significant regional deviation from the mean was expressed by a Z-score using a threshold value of Z >2.33 (p < 0.01, one sided) [17].

To supplement the individual evaluation of the PET images we performed voxel-based image analysis using statistical parametric mapping software (SPM5, Wellcome Department of Cognitive Neurology, London, UK; http://www.fil.ion.ucl.ac.uk/spm/). The intent of this strategy was explorative. The underlying assumption was that a linear correlation exists in a regionally specific manner between cerebral activity in grey matter structures and demographic or serologic parameters.

In all subjects the complete PET brain volume was sampled. PET images were transformed into the standard stereotaxic atlas of Talairach and Tournoux [18] using the PET template from Montreal Neurological Institute, Canada (MNI). PET images were smoothed with a 16-mm FWHM isotropic Gaussian kernel. The differences in global activity were removed by ratio adjustment, estimating global activity independently of changes in local activity.

To identify statistically significant changes, repeated statistical analyses were carried out using the general linear model correlating a parameter of interest with regional cerebral activity. As the analyses are explorative and multiple non-independent comparisons are performed it is necessary to set a stringent threshold value that protects against false positive findings. A statistical threshold was used at p < 0.05 (FDR, false discovery rate) [19] corrected at the voxel level for multiple comparisons. The FDR has been validated and found useful for imaging studies. It controls the expected proportion of false positives among suprathreshold voxels. The parameters analyzed were years of known HIV infection, BDNF, CRP, nadir CD4 level, TNF, IL-6, suPAR, max. log. HIV RNA copies, and log transformed levels of BDNF, TNF, IL-6, and suPAR.

### Statistical analyses

Demographic and serological data were analysed using SSPS version 13.0 for Windows. Mean values of the various parameters were compared group-wise in independent sample *t*-tests. Pearson Chi-Square Test and Fischer's Exact Test s were used to assess potential correlations between mode of HIV transmission and abnormal PET scanning results. *P *values of < 0.05 were considered significant and *P *values between 0.05 and 0.099 considered to be of borderline significance. Data not normally distributed were log-transformed. Linear correlations were analysed with Pearson correlations.

### Ethical considerations

The project was approved by the Regional Committee on Biomedical Research Ethics of Copenhagen and Frederiksberg municipalities, reference number: (KF) 01-192/03.

## Results

**Figure 1:**
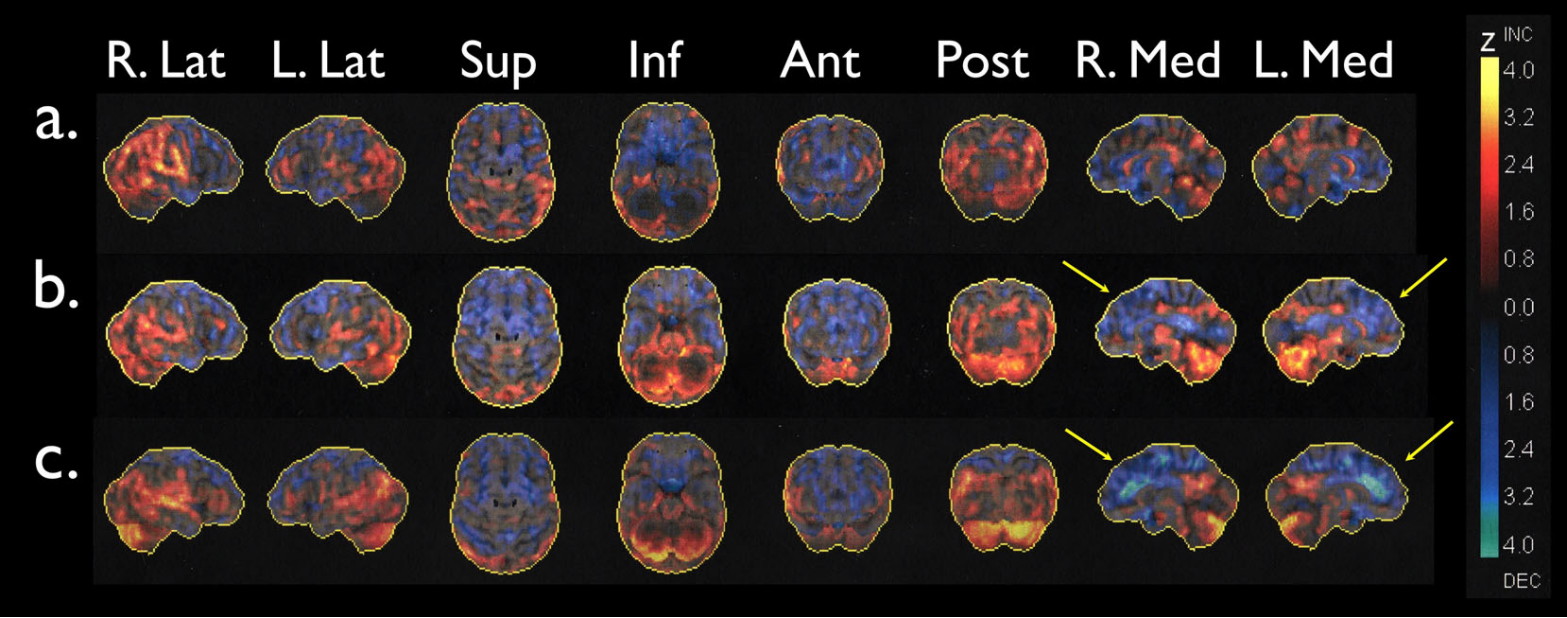
**Maximum Z-score maps from 3 individuals compared to a group of healthy controls using the Neurostat software**. Surface projections display the Z-score maps from lateral, superior, inferior, anterior, posterior, and medial views. The images are scaled from -4.0 to 4.0 in Z-scores with the warm colours representing areas that are more active than the control group, and the cold areas representing areas that are less active. Subject a) was evaluated as normal, subject b) as possibly abnormal, and subject c) as abnormal. The arrows point to significant reductions in the medial frontal cortices.

A cohort of 38 HIV infected patients, optimally treated with no virological failure for more than three years underwent cerebral FDG-PET scanning. The patients were carefully selected in order to include patients with HIV as mono-morbidity. The patients were quite experienced with known HIV infection for almost 12 years (range 4 - 21 years) (table 1). Twenty-six patients (68%) had part- or full time jobs and none had any overt neurological manifestations. However, 21 (55%) of their cerebral FDG PET-scanning images had focal abnormalities when compared individually to age-matched controls. These primarily consisted of metabolic reductions of varying severity in the mesial frontal cortices involving the anterior cingulated cortex (Figure 1). The mesial frontal lobes are targeted in a number of different neuronal pathologies. Thus, it is a prominent feature of neurodegenerative disease, particularly frontotemporal dementia (FTD) [20], and of neuropsychiatric systemic lupus erythematosus (SLE) [21].

**Table 1:** Characteristics of study participants (n = 38)

Sex (n)	
Males	32
Females	6
Ethnicity (n)	
Caucasian	35
African	2
African-American	1
Mode of HIV infection (n)	
Heterosexual	10
MSM^a^	38
IVDU^b^	0
Age	48.4 ± 9.2 (34-68)*
Years of known HIV infection	11.9 ± 4.9 (4-21)
Years on ARV therapy	7.7 ± 2.7 (3-16)
Years on didanosine and/or stavudine	1.5 ± 2.3 (0-7)
Nadir CD4 count (10^6 ^cells/l)	190 ± 188 (10-740)
Maximum HIV RNA (log copies/ml)^C^	5.2 ± 0.6 (3.6-6.4)

^a ^MSM: males having sex with males; ^b ^IVDU: intra-venous drug users; ^c^ data only available for 30 patients

* Data expressed as mean ± SD (range)

The correlates of the PET abnormalities are summarised in table 2. The PET scanning abnormalities were correlated with age, as the patients with abnormal PET scanning results were slightly older (mean age 49.9 years) than those with a normal PET scanning (mean age 46.6 years, p = 0.3). However, the age effects were controlled for in the Neurostat analysis by comparing patients with age-matched healthy controls. The patients with abnormal cerebral FDG-PET scanning had been aware of their HIV infection for fewer years (10.3 vs. 13.8 years, p = 0.03) and had received ARV treatment for a shorter time period (7 vs. 8.5 years, p = 0.08) than the patients with normal FDG-PET cerebral brain scanning results. None of the patients had any reported IV drug abuse (which would have excluded them from the study) and there was no correlation between mode of HIV transmission and an abnormal PET scanning result (Person Chi Square and Fischer's Exact Test *p *= 0.3).

**Table 2:** Demographics, HIV history and FDG-PET scanning results

	Normal PET n = 17	Abnormal PET n = 21	*p*-value^a^
	Mean ± SD	Mean ± SD	
Age (y)	46.6 ± 8.4	49.9 ± 9.8	0.3
Years of known HIV infection	13.8 ± 5.2	10.3 ± 4.2	0.03*
Nadir CD4 level (10^6 ^cells/l)	134 ± 144	236 ± 210	0.1
Max. log. HIV RNA copies/ml^b^	4.97 ± 0.7	5.34 ± 0.6	0.12
Years on ARV treatment	8.5 ± 3.3	7 ± 2	0.08*
Years on didanosine and/or stavudine	1.3 ± 1.9	1.7 ± 2.7	0.6

^a^*p *values, equal variance of means assumed, ^b ^data only available for 30 patients

*p-values < .05 considered significant, values between .05 -.099 considered borderline significant

No difference in exposure to potential mitochondriotoxic agents like didanosine and/or stavudine could be demonstrated. The patients had received a variety of ART regimens for various time spans over the years, thus not allowing comparison of neuropenetrating regimens with less neuropenetrating regimens. The mean nadir CD4 count of the group with normal PET scanning was lower than in the group with abnormal PET scans, but this difference was not statistically significant (p = 0.1). We would have expected a lower CD4 nadir in the group exhibiting PET abnormalities but noted that the nadir levels were quite low in both groups. The patients with FDG-PET scanning abnormalities had higher levels of circulating TNF α (p = 0.08) and IL-6 (p = 0.08) but no differences were observed in the levels of BDNF or suPAR (table 3). In bivariate analyses the suPAR and TNF α levels correlated with a Pearson coefficient of 0.29 (*p *= 0.08), but no correlation was observed among the other parameters measured. The SPM analyses correlating demographic and serological parameters to regional cerebral grey matter activity did not reveal any statistically significant findings. This widely used technique is designed to identify a *common *pattern of variation within or between groups. The lack of significant findings may reflect a combination of a relatively weak signal change and heterogeneity in the pattern of affected cortical regions.

**Table 3:** Plasma cytokine and soluble mediator levels and FDG-PET scanning results

	Normal PET n = 17	Abnormal PET n = 21	*p*-value^a^
	Mean ± SD	Mean ± SD	
BDNF ng/ml	5.3 ± 4.5	4.2 ± 3.6	0.4
TNF pg/ml	0.8 ± 0.4	1.1 ± 0.6	0.08*
suPAR ng/ml	3.7 ± 0.7	4.2 ± 1.1	0.1
IL-6 log pg/ml	0.07 ± 0.4	0.26 ± 0.3	0.08*

^a ^p values, equal variance of means assumed. *p-values < 0.05 considered significant, values between 0.05 and 0.099 considered borderline significant.

## Discussion

The patients included in this study were carefully screened for co-morbidities and fulfilled criteria for optimal ARV treatment: fully suppressed viral loads in plasma and a CD4 level above 200 × 10^6 ^cells/l for a minimum of three years. In fact, the mean number of years on ARV was 7.7 for the entire group and more than 68% were working full- or part time at the time of the study. Surprisingly, half of these well functioning patients exhibited cerebral FDG-PET scanning abnormalities associated with increased levels of circulating TNF α and IL-6 (*p *= 0.08). Other studies have demonstrated an association between circulating levels of TNF α and IL-6 in neurodegenerative conditions [9,22]. However, these functional associations have been heterogeneous, and we could not from our data demonstrate a consistent pattern that systematically associated TNF α or IL-6 levels with a defined set of grey matter structures.

suPAR levels have previously been found to be increased in patients with ADC or opportunistic CNS infections [23,24]. The abnormalities described by FDG-PET scanning are probably only a very early sign of suffering neurons, and overt cell damage may not necessarily have occurred yet. This could explain why suPAR and BDNF levels were not correlated with the scanning results.

HIV 1 enters the central nervous system very early in HIV infection, and it is well established that HIV plays a role in late-stage disease with severe immuno-suppression and high viral loads. It is however still a matter of debate whether continued viral replication as measured in peripheral blood reflects conditions inside the blood-brain barrier (BBB) and, most importantly, whether it harms the brain [25-27]. Among the potential problems related to ARV therapy is the difference in drug concentrations achieved in the CSF compared to peripheral levels - leaving the CNS as a *sanctuary site*. Furthermore, some ARV drugs are toxic to the γ polymerase of mitochondria, rendering cells with low-level metabolic activity susceptible to this toxic impact [28]. It was not possible in this study to demonstrate a neurodamaging effect that correlated with use of these drugs. Several explanations may be thought of e.g. too small a sample size or too short an exposure.

A recent prospective MRI study reported signs of faster grey and white matter volume loss in HIV-positive patients (in adequately treated patients as well as in patients with detectable viraemia) compared to HIV-negative patients [29]. The authors do not offer an explanation but suggest that the cognitive function of HIV patients receiving ARV treatment should be monitored.

## Conclusion

A substantial fraction of optimally treated HIV patients exhibit metabolic abnormalities of cerebral glucose metabolism, which may represent imminent neuronal damage. This group of patients had been aware of their HIV infection and had been on ARV for fewer years than those patients without abnormalities. It could be speculated that these patients have suffered longer brain exposure to high HIV loads than those without abnormalities. The potential neuroprotective effect of early ARV treatment should be considered but further studies and prospective follow-up studies including detailed neuropsychological testing are needed to evaluate the clinical implications of these results.

## Competing interests

ABA has received fees from Bristol-Myers Squibb, Abbott and Merck Sharp & Dohme. AML has received fees from Abbott, Bristol-Myers Squibb and GlaxoSmithKline. JG has received research funding from Abbott, Roche, Bristol-Myers Squibb, Merk Sharp & Dohme, Pharmacia, GlaxoSmithKline, Swedish Orphan and Boehringer Ingelheim. IL, KSK, HB SRO LH and AK declare no conflicts of interest.

## Authors' contributions

ABA, IL, LH, AML, JG and AK designed the study. IL was responsible for and analyzed brain FDG-PET scanning analyses. KK, HB, SO and HU made the cytokine analyses. ABA was responsible for patient inclusion. All authors participated in manuscript preparation. All authors have read and approved the final manuscript.
